# Electromagnetic radiation driving of volume changes in nanocomposites made of a thermosensitive hydrogel polymerized around conducting polymer nanoparticles†

**DOI:** 10.1039/d0ra01329c

**Published:** 2020-03-03

**Authors:** Silvestre Bongiovanni Abel, Claudia R. Rivarola, Cesar A. Barbero, Maria Molina

**Affiliations:** Research Institute for Energy Technologies and Advanced Materials (IITEMA), National University of Rio Cuarto (UNRC), National Council of Scientific and Technical Research (CONICET) Ruta Nacional No. 36 Km 601, Agencia Postal No. 3 5800 Río Cuarto Argentina mmolina@exa.unrc.edu.ar +543584676233 +543584676522

## Abstract

Polymeric nanocomposites were obtained by the formation of a thermosensitive hydrogel matrix around conducting polymer (CP) nanoparticles. The CP is able to absorb electromagnetic radiation which is converted into heat and induces the phase transition of the surrounding hydrogel. The method chosen to form the hydrogel is the free radical polymerization of a copolymer (*N*-isopropylacrylamide (NIPAM) and 2-acrylamide-2-methylpropano sulfonic acid (AMPS), PNIPAM-*co*-2% AMPS) in the presence of bisacrylamide as the crosslinker. The nanoparticles are polypyrrole nanospheres (PPy NP), polyaniline nanofibers (PANI NF), and polyaniline nanospheres (PANI NP). The morphology of the composites was studied using SEM microscopy and the percentage composition of each material was evaluated by thermogravimetric analysis (TGA). The swelling equilibrium capacity and rate are clearly affected by the nanoparticle shape and nature. However, the nanocomposites LCST are similar to that of the matrix. Upon RF irradiation, the three nanocomposites increase the temperature and reach the LCST after 320 seconds of irradiation (320 kJ). Upon MW application, the local temperature reaches the LCST after only 30 s (21 kJ), resulting in a MW more effective than RF to drive the transition. These results demonstrate that the proposed materials are useful as a remotely driven actuator.

## Abstract 2

A novel synthetic method of photothermally activated nanocomposites by *in situ* formation of hydrogel matrixes around dispersed conducting polymer nanoparticles is described.

## Graphical abstract

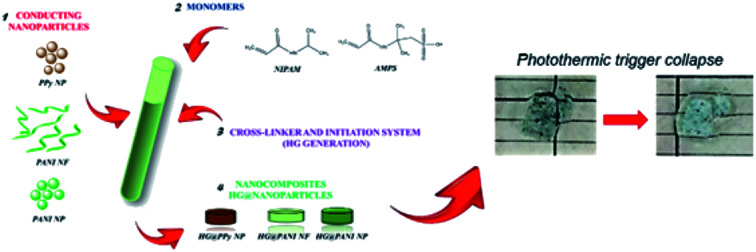

## Introduction

Hydrogels are physically or chemically crosslinked 3D networks with the capacity to absorb large amounts of aqueous solutions.^1^ “Smart hydrogels” are materials which suffer volume changes upon the application of external stimuli such as temperature, pH, electric field, magnetic force, *etc.*^2,3^ In particular, temperature-responsive hydrogels can swell or deswell upon heating or cooling. The polymers chains have a lower critical solution temperature (LCST), where a hydrophilic to hydrophobic transition occurs where the chains change from a coil to globule conformation. When the linear chains inside a crosslinked polymer (hydrogel) are exposed to a temperature above the LCST, the coil to globule transition of the linear chains induces a clear volume phase transition at a defined temperature (LCST). Below the LCST, the enthalpic component – related to hydrogen bonding between polar groups (*e.g.* <svg xmlns="http://www.w3.org/2000/svg" version="1.0" width="10.400000pt" height="16.000000pt" viewBox="0 0 10.400000 16.000000" preserveAspectRatio="xMidYMid meet"><metadata> Created by potrace 1.16, written by Peter Selinger 2001-2019 </metadata><g transform="translate(1.000000,15.000000) scale(0.011667,-0.011667)" fill="currentColor" stroke="none"><path d="M80 1160 l0 -40 40 0 40 0 0 -40 0 -40 40 0 40 0 0 -40 0 -40 40 0 40 0 0 -40 0 -40 40 0 40 0 0 -40 0 -40 40 0 40 0 0 -40 0 -40 40 0 40 0 0 -40 0 -40 40 0 40 0 0 80 0 80 -40 0 -40 0 0 40 0 40 -40 0 -40 0 0 40 0 40 -40 0 -40 0 0 40 0 40 -40 0 -40 0 0 40 0 40 -40 0 -40 0 0 40 0 40 -80 0 -80 0 0 -40z M560 520 l0 -40 -40 0 -40 0 0 -40 0 -40 -40 0 -40 0 0 -40 0 -40 -40 0 -40 0 0 -40 0 -40 -40 0 -40 0 0 -40 0 -40 -40 0 -40 0 0 -40 0 -40 -40 0 -40 0 0 -40 0 -40 80 0 80 0 0 40 0 40 40 0 40 0 0 40 0 40 40 0 40 0 0 40 0 40 40 0 40 0 0 40 0 40 40 0 40 0 0 40 0 40 40 0 40 0 0 80 0 80 -40 0 -40 0 0 -40z"/></g></svg> N–H on C=O) and water molecules – dominates the thermodynamic interactions and results in a swollen hydrogel.^4^ Above this critical temperature, the transition of the polymeric chains to globular form (interaction among the chains and not with the solvent) and the hydrogel volume collapses.^5,6^

Conducting polymers have been extensively investigated owing to their special electrical properties, which allow applying the materials in diverse fields: energy storage,^7,8^ sensors, actuators,^9,10^ drug delivery,^11,12^ biomedical applications,^13–15^*etc.*^16,17^ When this class of polymer is incorporated into a hydrogel, provide an excellent interface between the electronic-transporting and the ionic-transporting phases, and between soft and hard materials.^18^ Recent trends have focused on generating nanomaterials from conducting polymers. One of the advantages about incorporating nanomaterials in polymeric matrices is that could generate new advanced materials that exhibit superior physical, mechanical, thermal, and conductive properties.^19^ Nanomaterials can add the response of bulk materials (*e.g.* hydrogels) to a new stimulus, such as electromagnetic radiation which can be modulated in a versatile way.^1^ In this sense, a wide variety of nanomaterials based on conducting polymers has been generated: nanoparticles,^20,21^ nanofibers,^22^ nanowires,^23^ nanogels,^24^ thin films,^10^ and others.^25^

Polymeric composites are one specific type of composites in which polymer acts as the matrix to contain other components in the nanoscale which acts as the nanofiller.^26^ Different kinds of polymeric composites were explored in the last years.^27–29^ One of the more interesting systems is built when the physical, mechanical, and thermosensitive properties of hydrogels are combined with the electrical properties of the conducting polymers.^30^ Recently, several methods to obtain conducting polymer composites using hydrogels as the matrix were reviewed by Stejskal.^31^ Among the main strategies stand out some ways such as the preparation of the conducting polymer within the hydrogel matrix,^32,33^ penetration of hydrogel with the conducting polymer,^6,34^ cryogels,^35,36^ aerogels,^37,38^ unsupported hydrogels,^39^*etc.* However, to the best of our knowledge, the generation of conducting polymer nanocomposites starting from conducting nanoparticles dispersions and growing the thermosensitive matrix (hydrogel) around them has not been described.

The photothermal effect involves the absorption of electromagnetic radiation by a material followed by the release of that energy as heat, inducing an increase of the temperature of the irradiated material. One example is the absorption of Near Infrared Radiation (NIR) by a nanomaterial which generates localized heat. Some conducting polymer materials showed excellent performance by absorption of electromagnetic radiation.^40,41^ Microwaves (MW) were also used to heat polymeric composites based on a thermosensitive matrix with conducting or metallic nanofillers.^34^ The effect allowed the building of remote actuators activated by microwaves.^35^ Another kind of electromagnetic radiation employed to generate heat on materials is radiofrequency (RF), which is electromagnetic radiation at lower frequencies (kHz) than microwaves (MHz to GHz). Different reports have shown the possibility to produce Joule effect by molecular frictional agitation (RF) or rotation of water molecules under alternating electric field (MW) with the subsequent heat generation.^42,43^ However, to the best of our knowledge, the heating of nanocomposites induced by radiofrequency absorption using conducting nanoparticles was not reported previously. Modern power electronic circuits allow producing easily high power RF signals which are widely used in industry to seal by localized heating.^44^

Herein, we present a novel methodology to generate nanocomposites using conducting polymeric nano-objects and a thermosensitive matrix based on PNIPAM-*co*-AMPS – poly(*N*-isopropylacrylamide-*co*-2-acrylamide-2-methylpropano sulfonic acid) – following a bottom up approach. The new way to generate composites involves the formation of a thermosensitive matrix in the presence of previously synthesized conductive nanoparticles dispersions. The principal advantage of the synthetic process is the possibility of working only with aqueous solutions, without the need to count with a structure of controlled porosity for nano-objects incorporation. Moreover, the nanomaterial can be completely characterized before nanocomposite synthesis. Besides, since the conducting polymers and the hydrogel are separate phases, the LCST of the materials should not be affected by the incorporation of the conducting polymers in several forms (nanofibers or spherical nanoparticles). Since conducting polymer nanoparticles have been shown to efficiently absorb electromagnetic radiation to induce a local temperature change,^20^ they could be used to produce photothermally activated nanocomposites. The induced volume phase transition of the thermosensitive matrix provoked by the photothermal effect could be advantaged to the remote release of a drug with a specific function. The kind of source irradiation could be chosen depending on the desired application.

## Experimental

### Materials

Anilinium hydrochloride (ANI·HCl), aniline (ANI), and pyrrole (Py) monomers used to produce nanofibers and nanoparticles were obtained from Merck (ANI·HCl) and S. Aldrich, respectively. The chosen nanoparticles stabilizer was polyvinylpyrrolidone (PVP, Fluka, type K90, *M*_w_ = 360 000). For composites generation, *N*-isopropylacrylamide (NIPAM) and 2-acrylamide-2-methylpropano sulfonic acid (AMPS) monomers were purchased from Scientific Polymer Products. *N*,*N*-Methylenebisacrylamide (BIS) from Sigma-Aldrich was used as crosslinker agent. Initiator system of polymerization: ammonium persulfate (APS) and *N*,*N*,*N*′,*N*′-tetramethylenediamine (TEMED), obtained from Sigma-Aldrich. Water was triply distilled. All other reagents and solvents in this work were used as received and are analytical quality.

### Synthesis

#### Nanoparticles

Before the generation of the composites, conducting polymer nanoparticles were synthesized using known methods.

Polypyrrole spherical nanoparticles (PPy NP) were obtained by a modification of the method described by Woo *et al.*^45^ Synthesis was carried out using APS (0.08 M) as an oxidant of the aqueous solution of monomer (Py, 0.043 M). To stabilize the obtained nanoparticles, an aqueous solution of PVP (1% w/w) was used. The reaction of polymerization was carried out for 30 minutes at room temperature.

Polyaniline nanofibers (PANI NF) were produced using the method reported by Kaner and co-workers.^46^ The monomer (ANI, 24 mmol) was dissolved in an organic solvent (75 mL, chloroform) and ammonium peroxydisulfate (6 mmol) was dissolved in an aqueous solution of hydrochloric acid (75 mL, 0.8 M). A liquid–liquid interface was generated and the polymerization of the monomer to obtain nanofibers occurred during 24 hours in the dark. The low stationary concentration of aniline at the interface is soluble in the organic and the acidic aqueous media, while persulfate ion is only soluble in water. Therefore, the aniline monomer diffuses from the organic to the aqueous media, is oxidized and polymerizes. The polymer remains in the aqueous media. The controlled diffusion of aniline to the aqueous media creates polyaniline nanofibers. Finally, the aqueous phase containing the nanoparticles was collected.

Polyaniline nanospheres (PANI NP) were synthesized by precipitation polymerization from a solution of ANI·HCl 0.2 M in water, using APS (0.25 M) as the oxidant in presence of 2% w/w stabilizer (PVP) in water, as previously reported by Stejskal and coworkers.^25^ Polymerization was carried out at room temperature for 30 minutes and finally, a green dispersion of nanoparticles was obtained. The PANI NP dispersion was fully characterized in a previous work.^47^

#### Nanocomposite formation

**Fig. 1:**
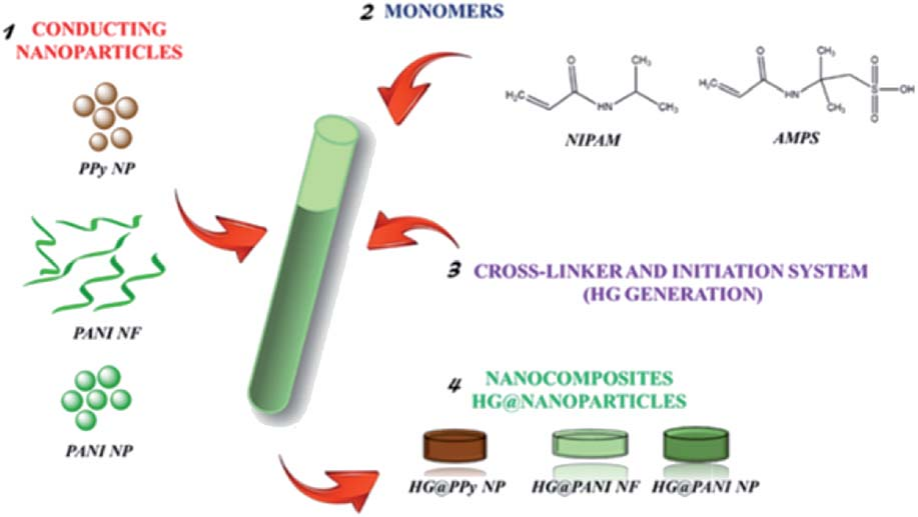
Nanocomposites generation representation using different kinds of conducting nanoparticles.

Nanocomposites were synthesized by free radical polymerization of NIPAM (0.5 M) with AMPS (in a molar ratio 98 : 2), using BIS as crosslinker agent (2% of total moles). Both monomers were dissolved in diluted dispersions (0.1 mg mL^−1^) of each nanomaterial (PPy NP, PANI NF, and PANI NP) as can be seen in Fig. 1. Then, the polymerization initiator system (APS (0.01 g mL^−1^) and TEMED (10 μL mL^−1^)) was added (0.001 g mL^−1^ and 10 μL mL^−1^, respectively). The crosslinked hydrogel grows around the nanoparticles, trapping them in the matrix. The nanoparticles of conducting polymer are preformed and no monomer (pyrrole or aniline) is present during the radical chain polymerization. Moreover, the persulfate produces radical species (together with TEMED) not acting as oxidant. The composites were obtained after 30 minutes of reaction at 20 °C. After the polymerization process, nanocomposites were purified by immersion in distilled water at room temperature for 48 hours, renewing the water several times to remove unreacted chemicals. It is important to highlight that the presence of nanoparticles influence negatively the gel formation, thus the concentration of nanoparticles should be kept low.

### Material's characterization

#### Scanning electron microscopy (SEM)

**Fig. 2:**
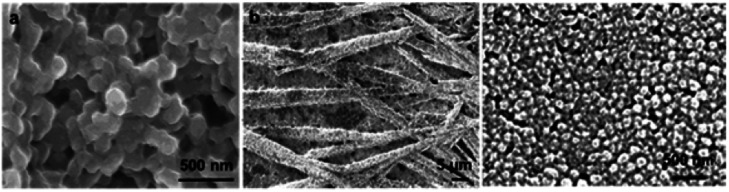
Scanning electronic micrographs of the different conducting nanoparticles used to generate the composites: (a) PPy NP, (b) PANI NF, and (c) PANI NP.

**Fig. 3:**
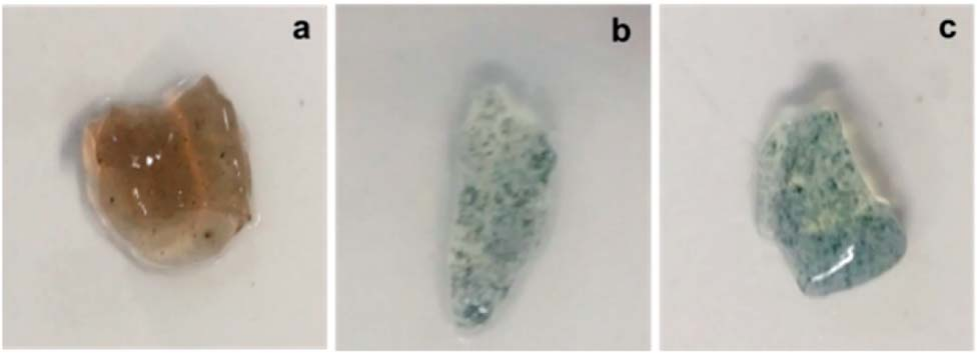
Macroscopic photographs of synthesized nanocomposites: (a) HG@PPy NP, (b) HG@PANI NF, and (c) HG@PANI NP.

Samples of nanoparticles (Fig. 2) and resulting composites (Fig. 3) were analyzed in a SEM at low vacuum and low field. A JEOL JSM6460 LV SEM was used to take the micrographs. The different samples were dried under vacuum (50 °C) to remove water in excess and then deposited onto a conductive grid for analysis. The observation was made after gold sputtering.

#### Thermogravimetric analysis (TGA)

Thermogravimetric measurements were taken in a Netzsch TG-209-F1-Libra 1 device. Quantities between 8–10 mg of dried nanocomposites were weighed in the quartz balance of the instrument. Samples were heated using a rate of 5 °C min^−1^ from room temperature to 500 °C under high purity N_2_ flow. Experimental error of these determinations was estimated in ±0.5% in weight loss measurement and ±2 °C respect temperature measurement.

#### Differential calorimetry scanning (DSC)

A Netzsch instrument DSC-204-F1 Phoenix device was used to perform the experiments of DSC. All the measurements were made under a high purity N_2_ atmosphere. Scanning was carried out from −30 °C to 50 °C using liquid N_2_ to cool the system. A heating rate of 3 °C min^−1^ was chosen. The experimental error in this assay was ±150 J g^−1^ and ±2 °C. Lower critical solution temperatures (LCST) were determined after DSC curves analysis.

#### Swelling kinetics and swelling rate constant calculation

Experiments were made using deionized water as solvent at room temperature. Dried pieces of each composite and hydrogel matrix without NP, previously washed and weighed, were placed in contact with the aqueous medium. At certain time intervals, samples were removed and weighed in an analytic balance and placed back into the bath. Each experiment was repeated until achieving a constant weight of composite disc (gravimetric method).

To calculate the swelling percentage (% Sw) as a function of time, eqn (1) was employed.1
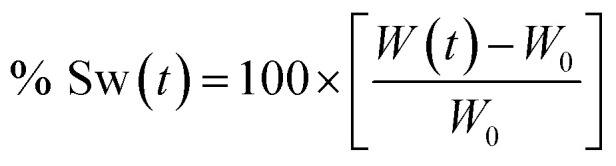
where *W*(*t*) represents the weight of composite in swollen state at time *t* and *W*_0_ is the weight of dry material.

Every data on the plot % Sw *vs.* time was obtained as the average of three individual measurements. Finally, using a first order adjust, the value of the swelling rate constant can be obtained.

#### Radiofrequency (RF) irradiation

The composites capacity to heat under exposition to radiofrequency was evaluated. Small pieces of composite hydrogels (*ca.* 1.5 g) were placed into glass Petri dishes and irradiated using a RF generator shown in Fig. 1 ESI.† The RF generator was a YNB Xiamen induction sealing apparatus which works at 30 kHz of frequency and up to 1200 W of output power. This frequency is in the range for biomedical applications, since the human tissue is transparent in that frequency range.^48^ Experiments were made during 420 seconds, taking photographs at initial and final states. The temperature of each material was measured on the surface with an infrared thermometer (TES 1326 s/1327K).

#### Microwave (MW) irradiation

Heat generation by microwave absorption was evaluated using a commercial microwave oven (Tyrrell) with the following technical characteristics: 2.4 GHz, 700 W nominal power, and medium power setting. Swelled composites pieces of *ca.* 1.8 g were placed on cover glass. The application time was 30 seconds in each case. The temperature was registered on the surface by TES 1326 s/1327K infrared thermometer. With the objective of contrast the initial and final states, optical photographs were taken. The same procedure was made with the control sample (PNIPAM-*co*-2% AMPS hydrogel).

## Results and discussion

### Materials morphology and composition

PANI NP, PANI NF, and PPy NP were prepared as described before. As it can be seen in Fig. 2, the expected shape of the nanoparticle was obtained in each case, spherical NP for PPy and PANI (Fig. 2a and c) and nanofibers for PANI NF (Fig. 2b). The full characterization of these nanoparticles has been reported previously.^49,50^

Using the aqueous dispersion of nanoparticles as polymerization medium, the composites were synthesized by the formation of hydrogels around the nanoparticles. The generated composites were characterized in morphological and compositional aspects. Fig. 3 shows the macroscopic photographs of the three types of generated composites. It is noteworthy that the coloration agrees with the color of nanoparticles dispersion: brown in the case of PPy NP, and green for materials based on PANI (NF and NP). Macroscopically, it is possible to observe the darker zones of agglomerated NP (more evident in the case of PANI), which were confirmed by SEM images. While there is some aggregation, the materials are uniform since no precipitation occurs during polymerization. It is required to use nanoparticles or nanofibers to produce stable dispersions where the hydrogel matrix could be produced.

The composites generation is proven by the fact that the formation of the hydrogel in presence of the dispersed nanoparticles gives rise to a solid with the coloration of the nanoparticles and the nanoparticles do not separate or are expelled from the matrix during washing or drying/swelling. It should be remembered that transmission electron microscopy (TEM) gives poor contrast because both the nanoparticles and the hydrogel are made of low atomic number elements (C, N, O, H). This is different from nancomposites of metal nanoparticles where good contrast is easy to get due to the large difference of atomic number.^51^ The situation is analogous at the formation of nanocomposites by gelation around carbon nanoparticles (nanotubes, graphene plates). However, it is possible that nanoparticles aggregate during gelation. Such phenomena seem to do not hinder the transfer of heat to the matrix, which induces the phase transition.

**Fig. 4:**
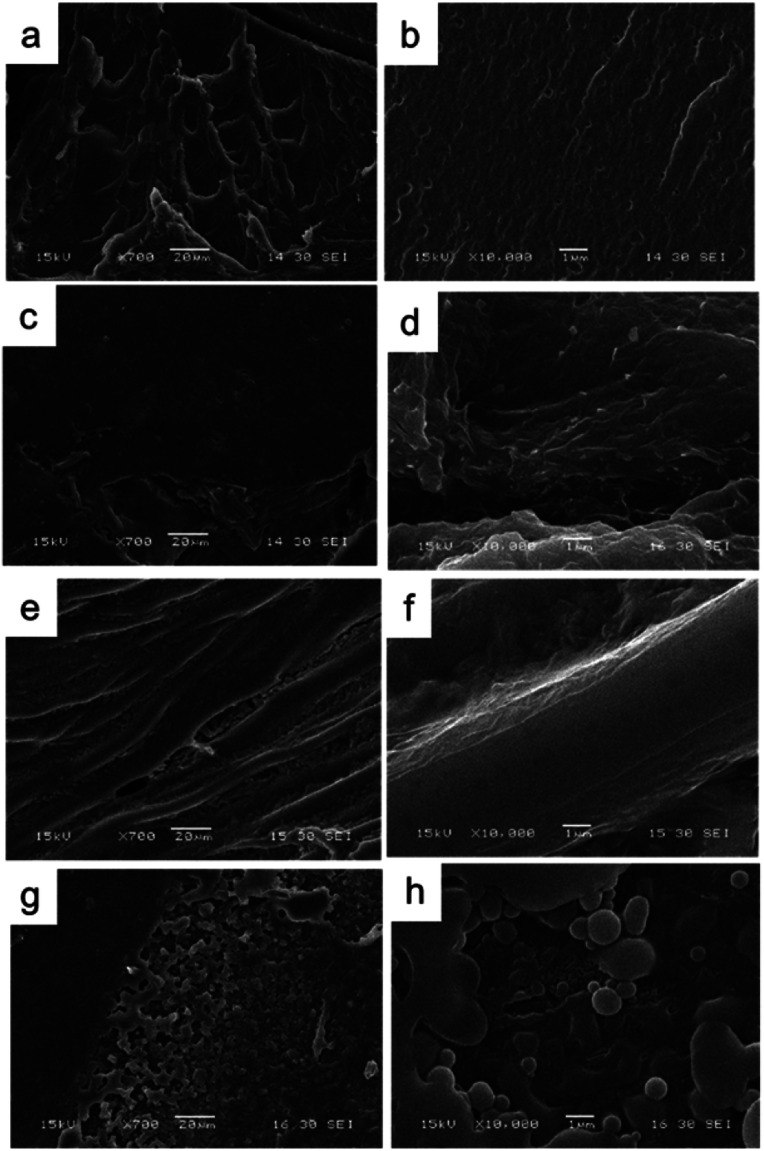
Scanning electronic microscopy images of the bare hydrogel and the obtained nanocomposites: (a) and (b) bare HG; (c) and (d) HG@PPy NP; (e) and (f) HG@PANI NF; (g) and (h) HG@PANI NP. Scale bar left 20 μm and right 1 μm.

As can be seen in Fig. 4, comparing the bare HG with the composites it is evident the presence of agglomerated nanoparticles in the composites. However, the SEM method (dry-state), show homogeneously distributed aggregates inside the thermosensitive matrix (PNIPAM-*co*-2% AMPS hydrogel). It is also possible to observe the difference of the network structure when polymerized in the absence or presence of the nano-objects. In Fig. 4c, d, g and h evidence of agglomerated spherical nanoparticles (HG@PPy NP and HG@PANI NP, respectively) can be observed. On the other hand, in the case of HG@PANI NF, it is possible to appreciate in Fig. 4e and f the presence of fibrillar structure of the network due to the incorporation of nanofibers inside the hydrogel matrix.

**Fig. 5:**
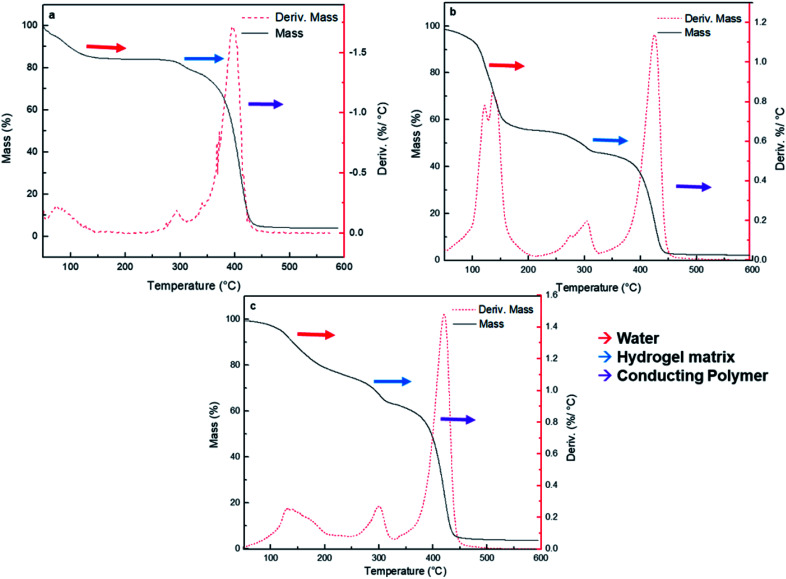
Thermogravimetric analysis of composites (black solid line) and loss weight *versus* temperature (red dash line) for each nanocomposite type: (a) HG@PPy NP, (b) HG@PANI NF, and (c) HG@PANI NP. Identification of water (red), hydrogel matrix (blue), and conducting polymer (purple) are highlighted in each case.

Thermogravimetric analysis (TGA) has been used to analyze the weight loss of the nanocomposites by thermal degradation.^52^ TGA assays of the synthesized HG@PPy NP, HG@PANI NF, and HG@PANI NP were made and shown in Fig. 5a, b and c, respectively.

Three major stages of weight loss are observed in all cases. Lost of weight at 90–100 °C could correspond to retained water in the materials: HG@PPy NP the (*ca.* ∼20%), HG@PANI NP (∼20%) and HG@PANI NF (∼40%). It is noteworthy that such water content is related to the water adsorbed in the free surface of collapsed gels which has been dried at 50 °C and it is only lost after the gel is heated at 90–120 °C. Such adsorbed water is not directly associated to the water absorbed during swelling. It could be related to the larger exposed area of conducting polymer nanoparticles in the “dry” state. At higher temperatures, a peak at ∼300 °C appears. It can be attributed to the degradation of the hydrogel matrix (PNIPAM-*co*-2% AMPS) by the imine formation from amide groups and the thermal degradation of the lateral hydrophobic chains.^53^ In all cases, ∼10% of hydrogel matrix composition is present in the nanocomposites. The third stage happens in the range of 350–450 °C and represents a 40–60% of weight loss due to the decomposition of amide groups and the degradation of the polymer main chains.^54^ On the other hand, it was reported that the degradation and the weight loss of PANI materials take place in three stages. The evaporation of moisture and impurities is observed up to 100 °C (10%); the second weight loss from 140 to 435 °C (15%) is due to the structural decomposition of the conductive polymer and PVP (stabilizer). For pure PANI, the major weight loss occurs after 435 °C indicating the large-scale structural decomposition of the polymer.^55,56^ Also, it has been reported PANI degradation by two stages in the range from 30 to 150 °C (weight loss of small compounds) and around 400 to 600 °C (degradation of polymer main chain).^57^ PPy degradation also occurs at three stages as PANI.^58^ First stage is observed at temperature 110 °C (10%), which is attributed to the expulsion of moisture in the polymer. In the second stage, the weight loss occurs at 250 °C (20%) due to the degradation of PPy. The last decomposition step initiates from 250 °C up to 1100 °C due to the release of C, H, and N moieties of PPy unit.^59^ According to the obtained TGA (Fig. 5), wide bands of derivate %/°C are not observed as described in the literature which could mean that it is not possible to detect the low percentage of NP (0.13% theoretical data) by this technique.

### Swelling kinetics in water

**Fig. 6:**
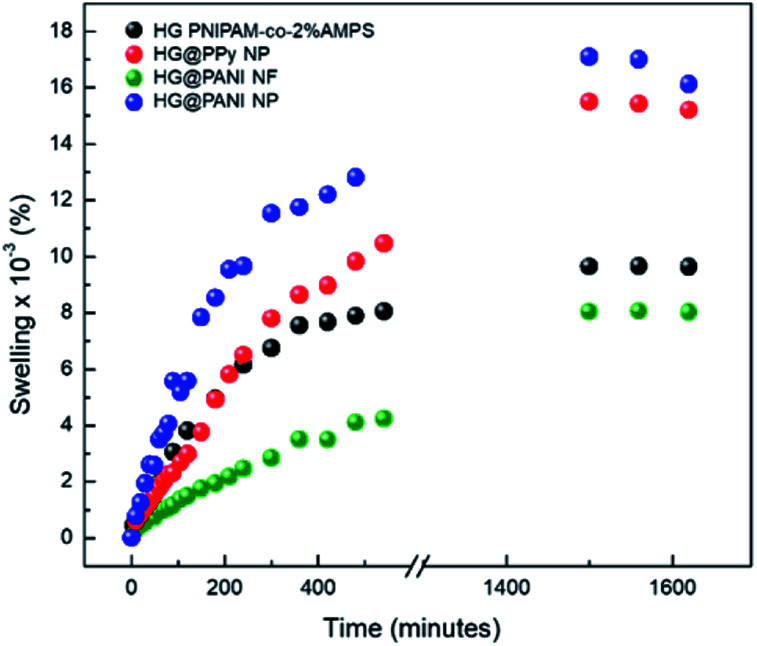
Swelling kinetics for each composite in aqueous solution at pH 7 and 25 °C.

In Fig. 6 are shown the swelling kinetics of the hydrogel matrix and nanocomposites in water at room temperature (25 °C) and pH 7. The equilibrium swelling percentage (% Sw_eq_) of the matrix involves the equilibrium between the driving force due to the solvation of free chain segments and the elastic force of the crosslinked matrix.^60^

This effect also affects the swelling rate constants (Table 1). In all cases, similar tendencies were observed for both parameters (% Sw_eq_ and swelling rate constants). The equilibrium swelling was reached after 1400 minutes (∼24 hours) of immersion in water. The higher swelling values were observed in the case of the HG@PPy NP composite (% Sw_eq_ = 16 100%) and for HG@PANI NP (% Sw_eq_ = 15 200%) than HG matrix (% Sw_eq_ = 9700%) without NP but HG@PANI NF shown a considerable decreasing of swelling in water (% Sw_eq_ = 8000%). While conducting polymers are less hydrophilic than PNIPAM-*co*-AMPS as shown by contact angle measurements,^61,62^ the composites show higher swelling indicating that it is not due to the contribution of NP to the hydrophilic properties (which should show opposite effect) but a change in the polymerization mechanism with a decrease of the crosslinking ratio due to the presence of the NP. Accordingly, the nanofibers which could crosslink the hydrogel at longer ranges due to its high aspect ratios show the smaller swelling. On the other hand, in the case of nanoparticles, the dominant effect is the decreasing degree of crosslinking due to the termination of chains by conducting polymer NP.

**Table 1:** Swelling rate constant for the different materials involucrate in this work

Material	Swelling rate constant (s^−1^) × 10^3^
HG PNIPAM-*co*-2% AMPS	3.96
HG@PPy NP	3.64
HG@PANI NF	1.05
HG@PANI NP	1.97

Evidently, the distribution of spherical nanoparticles inside matrix induces the breaking of intramolecular interactions among matrix chains increasing the swelling driving force and the swelling capacity. On the other hand, PANI nanofibers have a large aspect ratio (micrometric length *versus* nanometric diameter), which allow creating long-range crosslinks of the polymer matrix. In this way, the swelling capacity decrease for HG@PANI NF.

### LCST of the nanocomposites

It is well known that PNIPAM based hydrogels present a defined LCST at ∼32 °C.^61^ When PNIPAM is copolymerized with another hydrophilic co-monomer (PAMPS), this critical temperature can be increased.^60^ In particular, for the case of PNIPAM-*co*-2% AMPS, it was founded that the LCST is close to 36.5 °C. Previously, it was reported that the incorporation of conducting polymers can affect the LCST value, depending on the composite generation method used.^6^Table 2 summarizes the LCST measured by DSC for each kind of generated nanocomposite (Fig. 2 ESI†). Considering the experimental error, it can be seen that, the LCST of composites are similar to the LCST of the thermosensitive matrix. Therefore, neither the theoretical content of NP (0.13%) regarding hydrogel mass nor the contact points present between NP and matrix do significantly affect the LCST of matrices.

**Table 2:** Volume phase transition temperature (LCST) determined by differential scanning calorimetric for the different composite materials

Material	LCST (°C)
HG PNIPAM-*co*-2% AMPS	36.5 ± 0.5
HG@PPy NP	36.0 ± 0.5
HG@PANI NF	35.4 ± 0.5
HG@PANI NP	37.4 ± 0.5

### Effect of different electromagnetic radiations on the nanocomposite

Conducting polymers are able to absorb electromagnetic radiation and convert it into heat. The oscillating magnetic field induces eddy currents in the conductor which heat up the material by Joule effect. In this case, RF (30 kHz) and microwaves (2.4 GHz) were chosen as irradiation sources. Technical characteristics (*e.g.* irradiation power, spot area, *etc.*) of the electromagnetic radiation are described in Table 1 ESI.† Our interest in applying that kind of radiation on nanocomposites is based on the fact that RF and MW are not significantly absorbed by biological tissues. Therefore, the proposed composites could be heated at distance inside the human body, to use them as a drug delivery system or in photothermal anticancer therapy.

### Radiofrequency (RF) absorption

RF radiation has been frequently used to increase the temperature of magnetic or metallic materials.^63–65^ In the case of conductive materials, the oscillating electromagnetic field creates eddy currents inside the material which heat the material by Joule effect (since the material has a finite conductivity). Additionally, the oscillating magnetic field could interact with polar groups and the fast reorientation cause dielectric loss.^66^ The latter effect also occurs in the hydrogel, creating a background small temperature change.

**Fig. 7:**
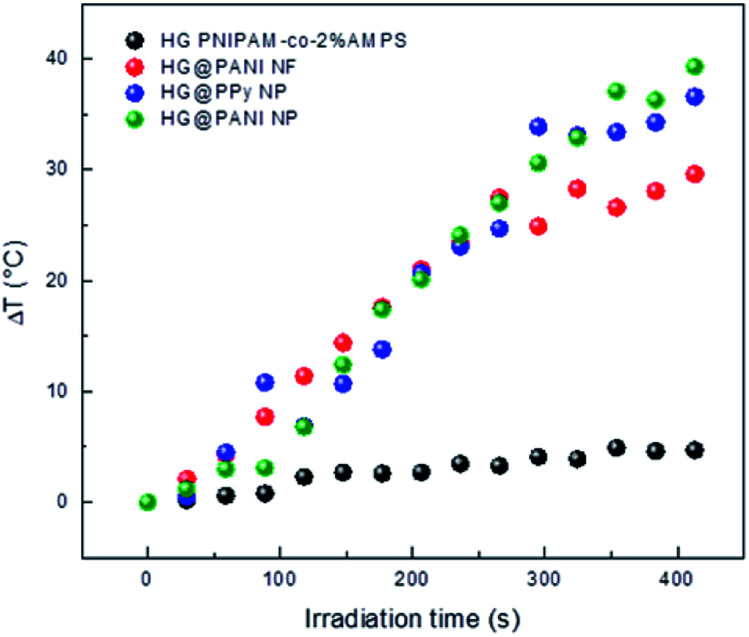
Temperature increment for each nanocomposite and the hydrogel matrix produced by radiofrequency (RF) irradiation measured in deionized water at pH 7. Initial temperature: 25 °C.

The effect generated on composites by RF irradiation is shown in Fig. 7. The results reveal that in the absence of conducting polymer, the temperature difference (Δ*T*) between initial time and after 420 seconds of irradiation is close to 3–4 °C. It is likely the small increase of temperature is due to that weak absorption due to the tail of the high frequency (17.4 GHz) absorption by water, which is related to dielectric loss.^67^ When the nanocomposites were exposed to RF, large increments of temperature were reached. For HG@PPy NP, Δ*T* was estimated to be 37 °C after the same irradiation time. In the case of HG@PANI NP a similar result was obtained (Δ*T* ∼ 35 °C), but for HG@PANI NF the temperature increment was smaller (Δ*T* ∼ 28 °C). The Δ*T* measured in the composites are large enough for biological applications.^68^ The energy applied to drive the transition can be calculated from the RF power and time to be 320 kJ.

**Fig. 8:**
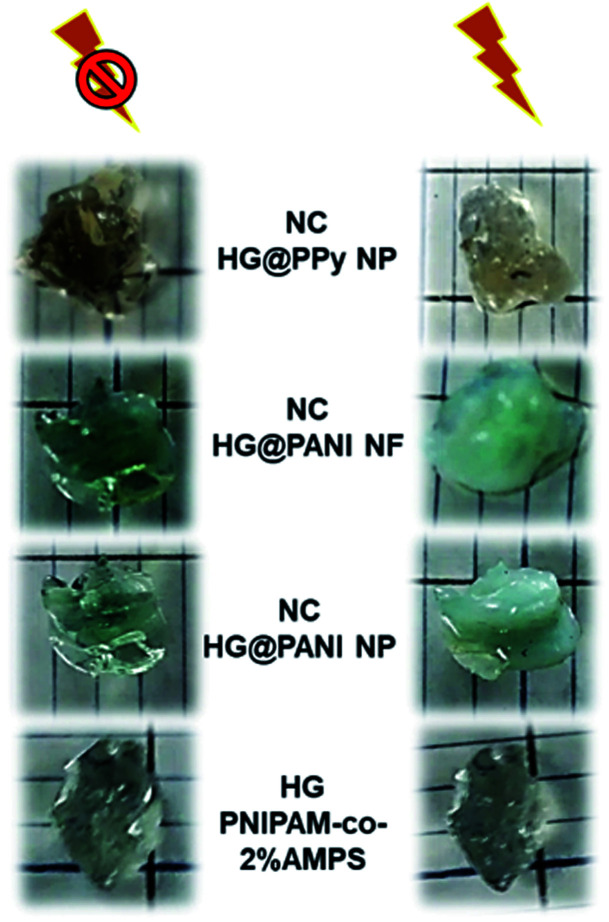
Collapse triggered after 420 seconds of radiofrequency (RF) irradiation for different nanocomposites. Comparison with a PNIPAM-*co*-2% AMPS hydrogel matrix without conducting polymer.

As can be seen in Fig. 8, the composite macroscopic aspect changes after the RF irradiation. It is clear that the increase of temperature induced by the irradiation triggers the phase transition of the thermosensitive matrix (PNIPAM). Note that the photographs of HG PNIPAM-*co*-2% AMPS alone (control) do not show changes upon the application of RF (in agreement with the results of Fig. 7) because the temperature is not higher than the LCST (due to lack of absorption). In summary, starting the RF irradiation at room temperature (25 °C) only 150 seconds (∼20 pulses), are enough to induce a Δ*T* > 10 °C and consequently the collapse of the materials.

### Microwaves (MW) absorption

**Fig. 9:**
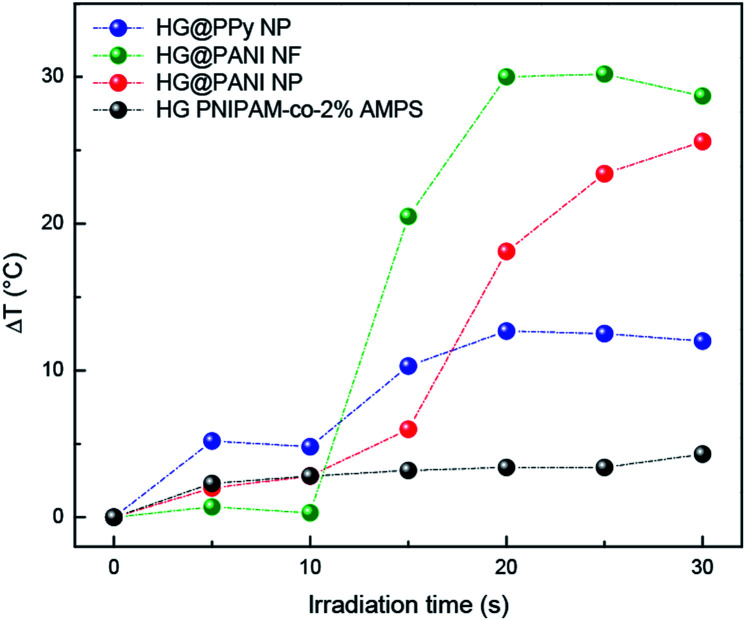
Temperature increment of each nanocomposite and hydrogel matrix produced by microwave (MW) irradiation. Initial temperature: 25 °C.

**Fig. 10:**
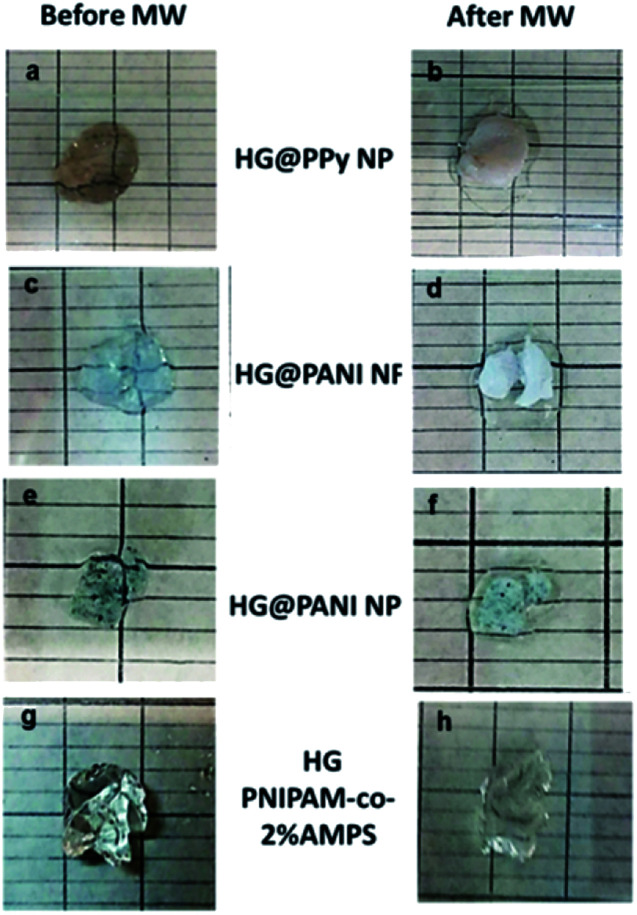
Macroscopic collapse evidenced after 30 seconds of microwave (MW) irradiation: (a) and (b) HG@PPy NP; (c) and (d) HG@PANI NF; (e) and (f) HG@PANI NP; (g) and (h) HG (PNIPAM-*co*-2% AMPS).

The same materials were studied using MW irradiation to heat up the nanocomposite (Fig. 9). After 30 seconds of irradiation, the hydrogel matrix showed only a Δ*T* < 5 °C due to the background absorption of the aqueous solution. The applied energy is only 21 kJ. On the other hand, the irradiation of HG@PPy NP induces a Δ*T* = 10 °C. In the case of the composites containing PANI, larger temperature changes were observed: Δ*T* ∼ 30 °C for HG@PANI NF and Δ*T* ∼ 25 °C for HG@PANI NP. In contrast with the reported results for radiofrequency irradiation, it is notable the fact that the time required to generate the phase transition by effect is much smaller in the case of using MW as irradiation source. In Fig. 10, the macroscopic changes due to the collapse of nanocomposites after MW irradiation can also be observed.

**Fig. 11:**
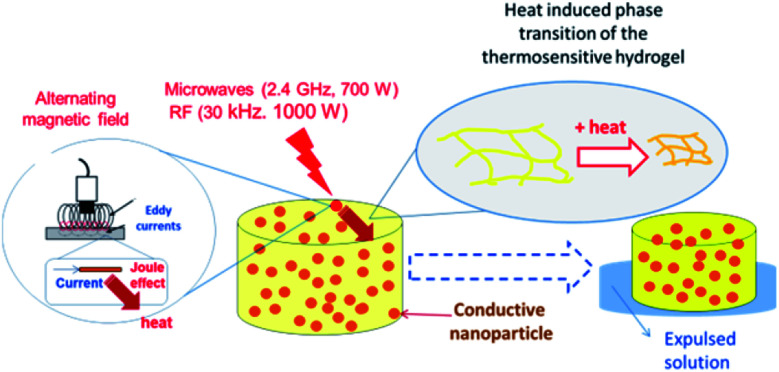
Mechanism of heat generation and hydrogel matrix volume change due to electromagnetic radiation (RF or MW) action.

The main mechanism of heating by RF or MW involves the generation of eddy currents in the conducting polymer nanoparticles (Faraday's 1^st^ law) which heat the material trough Joule effect (eqn (2) and Fig. 11).2
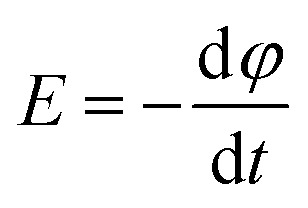
where *φ* is the magnetic field and *t* the time.

The induced voltage *E* will cause a current to flow that generates a magnetic field counteracting the change in the inducing field (Lenz's law). The conductor offers resistance to a flow of a current (*I*) which causes loss of power (*P*). The loss of power is converted to heat energy and is described in Joule's law:

3 *P* = *RI*^2^

where *R* is the resistance.

Conducting polymers have small conductivities (<1 S cm^−1^),^69^ therefore the heat loss is large, allowing to overcome the LCST of the thermosensitive polymer. Additionally, the dielectric loss of polar groups could contribute to the heat generation.

## Conclusions

A new kind of nanocomposites was synthesized by *in situ* formation of hydrogel matrices around dispersed conducting polymer nanoparticles. While the thermosensitivity is given by the PNIPAM based hydrogel matrix, the absorption of electromagnetic radiation is due to the electronic properties of the conductive polymer nanoparticles.

The swelling equilibrium capacity and rate are clearly affected by the nanoparticle shape. This is reasonable since the swelling is controlled by the hydration of the PNIPAM-*co*-2% AMPS chains (which is the same in all composites) counterbalanced by the elastic properties of the materials. The presence of nanofibers (PANI NF) makes the material more elastic due to a long-range mechanical fiber effect. On the other hand, nanospheres (PANI NP and PPy NP) could only make the composite less plastic due to the hindering of hydrogel chain movements.

The LCST of the nanocomposites is similar to the hydrogel matrix without nanoparticles, suggesting that a true nanocomposite is formed where the thermosensitive (PNIPAM) and conducting polymer (PPy or PANI) are present in two different phases. Two kinds of electromagnetic radiation – microwaves (2.4 GHz, 700 W) and radiofrequency (30 kHz, 1000 W) – are active to produce thermal effects that drive the collapse of the thermosensitive network (PNIPAM-*co*-AMPS). The applied energy required to induce the collapse is much smaller in the case of MW (21 kJ) than RF (320 kJ). The nanocomposites synthesized and characterized in this work are good candidates for drug delivery systems driven remotely using radiofrequency or microwaves, as it has been shown with similar nanocomposites using NIR light.^70^ This method of preparation, to the best of our knowledge, has not been used before to produce nanocomposites made of conducting polymer nanoparticles and hydrogel matrices. A comparison with other methods to produce the materials seems relevant and have been performed elsewhere.^71^

## Conflicts of interest

There are no conflicts to declare.

## Supplementary Material

RA-010-D0RA01329C-s001
