# Supplementary material for: Electromagnetic radiation driving of volume changes in nanocomposites made of a thermosensitive hydrogel polymerized around conducting polymer nanoparticles
Source: RSC Adv. 2020 Mar 3;10(15):9155–64. doi: 10.1039/d0ra01329c (PMC9050122; doi:10.1039/d0ra01329c)
Supplement: RA-010-D0RA01329C-s001 [file RA-010-D0RA01329C-s001.pdf]

Electronic Supplementary Material (ESI) for RSC Advances.
This journal is © The Royal Society of Chemistry 2020

Electromagnetic radiation driving of volume changes in nanocomposites made of a thermosensitive hydrogel polymerized around conducting polymer nanoparticles

Silvestre Bongiovanni Abel, [a,≠] Claudia R. Rivarola, [a] Cesar A. Barbero, [a] and Maria Molina [a,*]

[a] Instituto de Investigaciones en Tecnologías Energéticas y Materiales Avanzados (IITEMA), Departamento de Química, Universidad Nacional de Río Cuarto (UNRC)-Consejo Nacional de Investigaciones Científicas y Técnicas (CONICET). Ruta Nacional N° 36 Km 601, Agencia Postal N° 3, 5800, Río Cuarto, Argentina.

Supplementary Information

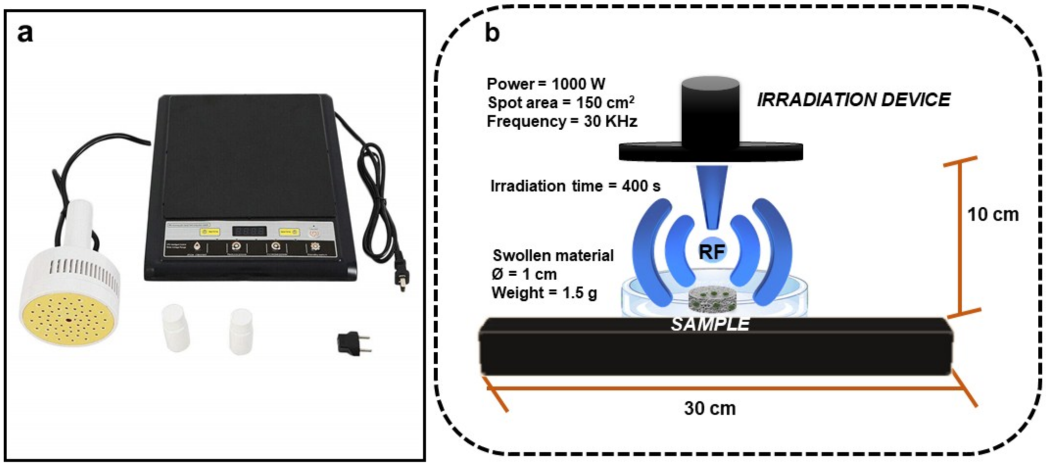

Figure 1 SI. a) Device used to perform the radiofrequency (RF) irradiation and b) diagram of the irradiation experiment.

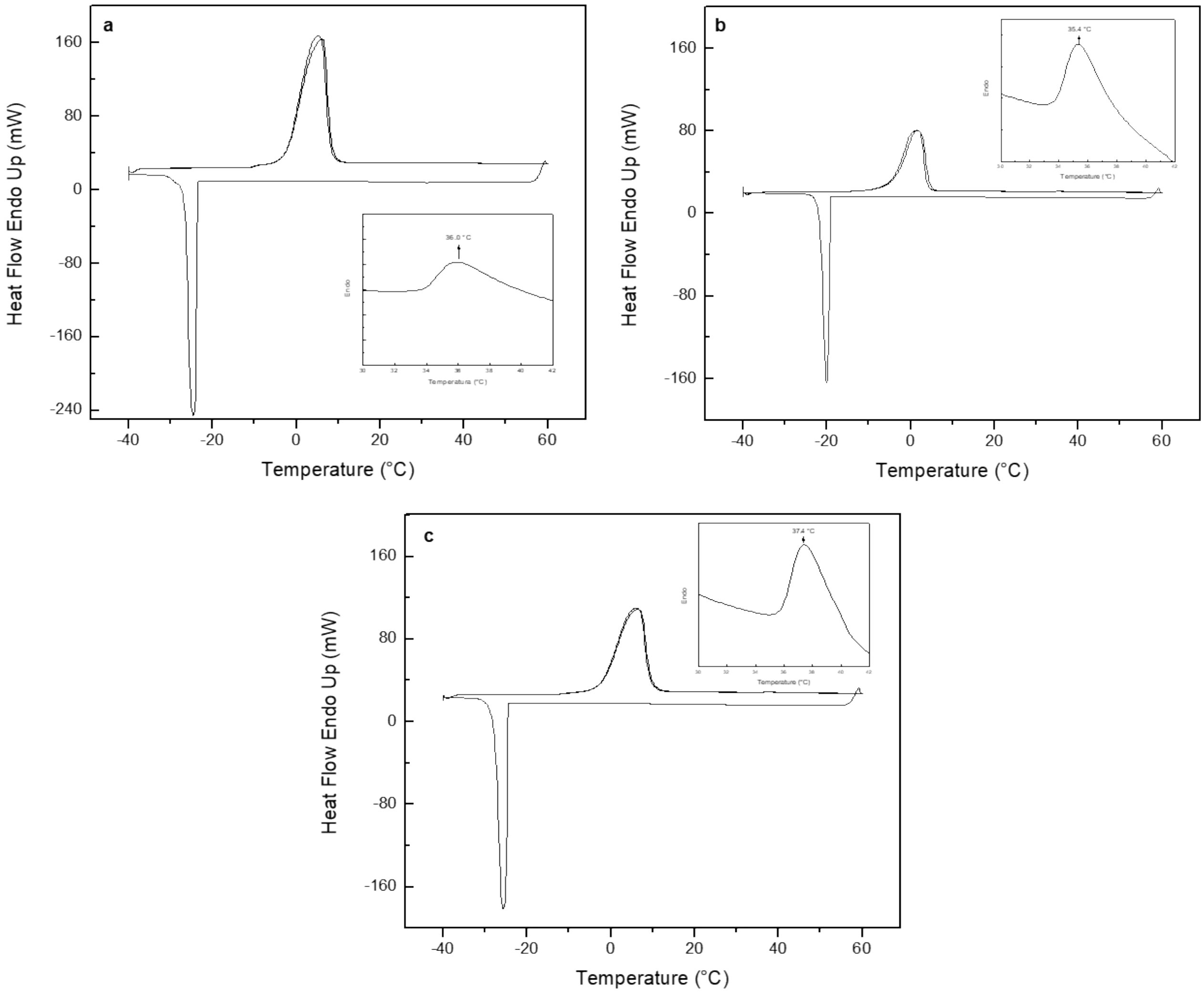

Figure 2 SI. Differential scanning calorimetric plots for: a) HG@PPy NP, b) HG@PANI NF, and c) HG@PANI NP.

Table 1 SI. Irradiation sources used to generate the photothermal effect on composites.

Electromagnetic radiation	Power (W)	Spot area (cm^2)
Microwaves (MW) 2.4 GHz	700	Whole
Radiofrequency (RF) 30 kHz	1000	150
